# Cluster of Lyme disease cases at a summer camp in Kent County, Maryland.

**DOI:** 10.3201/eid0201.960105

**Published:** 1996

**Authors:** G T Strickland, L Trivedi, S Watkins, M Clothier, J Grant, J Morgan, E Schmidtman, T Burkot

**Affiliations:** University of Maryland School of Medicine, Baltimore, Maryland, USA.


### Cluster of Lyme Disease Cases at a Summer Camp in Kent County, Maryland

Lyme disease is the second most prevalent emerging infectious disease in the United States; more than 65,000 cases have been reported to the Centers for Disease Control and Prevention since the disease was first described by Steere and colleagues in 1977 (1).

In July 1994, a physician in Chestertown, Maryland, reported eight cases of Lyme disease to the Kent County Health Department. Five were from a summer camp 10 miles north of Rock Hall on the Chesapeake Bay. In one case-patient, a 9-year-old camper from Pennsylvania, erythema migrans (EM) rash and left facial nerve palsy developed the day after she arrived at the camp.

To determine whether Lyme disease was present at the camp, we interviewed the eight counselors who had EM or febrile illnesses during July and 43 of the remaining 91 camp employees. Clusters of cases of Lyme disease with a short and specific exposure period (i.e., 10–12 weeks for the 100 counselors and 2–4 weeks for the 1,600 campers) had not been investigated in recent years.

All 51 surveyed camp employees gave histories of tick exposure throughout the summer. Four counselors had EM of 5 cm in diameter without other symptoms or signs and were treated with amoxicillin by the camp physician. Four other counselors had recurrent fever of 102°F to 104°F, severe headaches, somnolence, malaise, fatigue, myalgia, and anorexia. All four described extensive fatigue, drowsiness, and difficulty in getting out of bed. Three described shaking chills, and one had watery diarrhea. The camp physician admitted them all to the camp dispensary; Lyme disease was not diagnosed in any of them; only the patient with diarrhea was given an antimicrobial agent, trimethoprim/sulfamethoxazole. All patients improved in 3 to 5 days.

Sera were obtained in mid-August from the 51 employees; for the eight patients described above, this was 4 to 7 weeks after the onset of illness. All sera were nonreactive in indirect fluorescence antibody (IFA) tests against antigens for *Rickettsia rickettsii* and *Ehrlichia equi* (used to screen for human granulocytic ehrlichiosis). One patient, who had an EM-like rash but no other symptoms, had an IFA titer of 512 for *E. chaffeensis* (used to screen for human monocytic ehrlichiosis). Serologic testing for *Borrelia burgdorferi* by enzyme immunoassay (EIA) (Lyme Stat, BioWhittker, Walkersville, Maryland) identified patients with positive or borderline results (Table 1).

Hard ticks were collected by dragging felt material at several sites within the camp on three occasions during August. Collected adult *Ixodes scapularis* were tested by an antigen capture EIA for outer surface protein A (2). Ten (16.9%) of 59 male ticks were positive for *B. burgdorferi*. Although the infection rate was higher in female ticks collected from the camp, the results cannot be interpreted because the female ticks were cofed on rabbits; it is not certain whether this could cross-infect ticks feeding on the same animals.

We considered exposure to *B. burgdorferi* in this camp to be high (suspected acute Lyme disease-like illness incidence of 6% to 8%). The incidence rate depends on whether patients 6 and 7, who had flulike illnesses and positive EIAs and negative Western blot results (Marblot Strip Test System, Mardex Diagnostics, Carlsbad, California) are

Table. Results of WB antibody tests for *Borrelia burgdorferi* in summer camp residents with positive (titer ≥ 1.00) and borderline (titer = 0.80–0.99) EIA results

| | | Serology | | |
|---|---|---|---|---|
| | | | WB | |
| Subject | Syndrome | EIA | IgM | IgG |
| 1 | EM | 1.82 | Pos | Pos |
| 2 | EM | 1.40 | Neg | Pos |
| 3 | EM | 1.21 | Neg | Neg |
| 4 | EM | 3.21 | Pos | Pos |
| 5 | Flulike | 1.00 | Neg | Pos |
| 6 | Flulike | 1.04 | Neg | Neg |
| 7 | Flulike | 1.80 | Neg | Neg |
| 8 | Flulike | 2.46 | Neg | Pos |
| 9 | None | 0.96 | Neg | Neg |
| 10 | None | 0.96 | ND | Pos |
| 11 | None | 1.82 | Neg | Pos |
| 12 | None | 2.21 | Neg | Neg |
| 13 | Sinusitis | 1.11 | Pos | Neg |
| 14 | Sinusitis | 0.93 | Neg | Neg |
| 15 | None | 1.00 | Neg | Neg |
| 16 | Rocky Mountain spotted fever, 1991 | 1.14 | Neg | Neg |

EIA = enzyme immunoassay; EM = erythema migrans; ND = no data; WB = Western blot.

considered to have had Lyme disease, and on assuming that the 49 unexamined counselors did not have Lyme disease. Also, Kent County has one of the highest incidences of Lyme disease in the state (3), many deer were present in the woods and fields in and around the camp, and all counselors reported frequent exposure to ticks.

The four patients who had an acute febrile illness without cutaneous lesions were not initially suspected to have acute Lyme disease. We believe that flulike illness without EM is a more common manifestation of acute Lyme disease than is generally appreciated since, as in patients 5 through 8 (Table), Lyme disease is often not considered in the differential diagnosis (4). Acutely febrile patients, who have been bitten by a tick in Lyme disease-endemic areas also should be considered for early antibiotic therapy. Doxycycline or another tetracycline is effective for Lyme disease as well as for infections with *E. chaffeensis* and *R. rickettsii*, which are also transmitted by ticks and may have a similar clinical syndrome (5). Serologic testing, although it can confirm the diagnosis during the convalescence phase, may not establish an early diagnosis in either case, since antibody responses to all three infections are usually delayed until 2 to 4 weeks after the onset of symptoms and may not occur in patients treated with antibiotics (5-8).

We interpreted the Western blots according to criteria proposed at the Second National Meeting on Serological Diagnosis of Lyme disease (6). Of the four patients with EM and with EIAs positive for *B. burgdorferi*, patient 3, who had antibodies to *E. chaffeensis*, did not have IgG and/or IgM evidence of *B. burgdorferi* infection by Western blot. He also had no symptoms compatible with monocytic ehrlichiosis. Two (patients 6 and 7) of the four with flulike illnesses and with EIAs positive for *B. burgdorferi* did not have *B. burgdorferi* infection confirmed by Western blot. Positive or borderline serologic results for *B. burgdorferi* infection in patients 9 through 16 (Table) who did not have a clinical history compatible with Lyme disease could have been caused by asymptomatic infection, antibody responses from prior infections, cross-reactions from other infections, or false-positive reactions (8). Many of the counselors had been at the camp during previous summers and could have had prior mild, nondiagnosed infections with *B. burgdorferi*. Another possibility is that the EIA titers in some of the patients were high normal values, which may have been the case for patients 9, 14, 15, and 16. Patient 13 who had IgM evidence of recent infection on Western blot may have had a mild infection with *B. burgdorferi* during the previous month. However, this is impossible to confirm without acute-phase and convalescent-phase (or preexposure and postexposure) serum samples. This is also pertinent to those with flulike symptoms and negative Western blot results (patients 6 and 7).

The usefulness of using EIA screening and Western blot confirmation in seroepidemiologic studies for Lyme disease has not been established. The positive predictive value of a diagnostic test is highly dependent on the prevalence of the disease being studied. If the prevalence of Lyme disease in the population screened is very low, the positive predictive value of testing may be too low to be diagnostically useful.

**G. Thomas Strickland, M.D., Ph.D.,* Leena Trivedi, Ph.D.,† Stanley Watkins, B.S.,* Margaret Clothier, R.N.,‡ John Grant, M.D., M.P.H.,‡ John Morgan, M. D.,§ Edward Schmidtman, Ph.D.,¶ Thomas Burkot, Ph.D.#**

*University of Maryland School of Medicine, Baltimore, Maryland, USA; †Maryland Department of Health and Mental Hygiene, Baltimore, Maryland, USA; ‡Kent County Health Department, Chestertown, Maryland, USA; §Private practice of medicine, Chestertown, Maryland, USA; ¶U.S. Department of Agriculture Animal Research Station, Laramie, Wyoming, USA; #Division of Vector-Borne Infectious Diseases, National Center for Infectious Diseases, Centers for Disease Control and Prevention, Fort Collins, Colorado, USA.

### Acknowledgements

Dr. James Olson, Viral and Rickettsial Diseases Branch, National Center for Infectious Diseases, Centers for Disease Control and Prevention, performed IFA testing for *R. rickettsii and E. chaffeensis* antibodies. Dr. J. Stephen Dumler, Pathology Department, University of Maryland School of Medicine, performed the IFA testing for *E. equi* antibodies. We also thank Mr. Charles Hayword and his camp staff, including Ms. Carol M. Brown, for assisting in this investigation and Dr. Ebenezer Israel, Maryland Department of Health and Mental Hygiene, for advice and assistance. This project was supported by the Epidemiology and Disease Control Program, Maryland Department of Health and Mental Hygiene, and by the Agency for Health Care Policy and Research Grant 5 RO1 HSO7813.

### References

1. Steere AC, Maliwista SE, Snydman DR, Shope RE, Andiman WA, Ross MR, Steele FM. Lyme arthritis: an epidemic of oligoarticular arthritis in children and adults in three Connecticut communities. Arthritis Rheum 1977; 20:7-17.

2. Burkot TR, Patrican L, Piesman J. Field trial of an outer surface protein A (OspA) antigen-capture enzyme-linked immunosorbent assay (ELISA) to detect *Borrelia burgdorferi* in *Ixodes scapularis*. Am J Trop Med Hyg 1994; 50:354-8.
3. Steinberg SH, Strickland GT, Pena C, Israel E. Lyme disease surveillance in Maryland, 1992. Ann Epidemiol 1996; 6: In press.
4. Feder HM Jr., Gerber MA, Krause PJ, Ryan R, Shapiro Ed. Early Lyme disease: a flu-like illness without erythema migrans. Pediatrics 1993; 91:456-9.
5. Dumler JS, Bakken JS. Ehrlichial diseases of humans: emerging tick-borne infections. Clin Infect Dis 1995; 20:1102-10.
6. Recommendations for test performance and interpretation from the Second National Conference on Serologic Diagnosis of Lyme Disease. MMWR 1995; 44:590-1.
7. Engstrom SM, Shoop E, Johnson RC. Immunoblot interpretation criteria for serodiagnosis of early Lyme disease. J Clin Microbiol 1995; 33:419-27.
8. Magnarelli LA. Current status of laboratory diagnosis for Lyme disease. Am J Med 1995; 98:10S-14S.
